# The effect of cold-water mouth swilling on thermal perceptions and heat-related symptoms for people with multiple sclerosis exercising in a hot environment

**DOI:** 10.1007/s00421-025-05766-6

**Published:** 2025-04-05

**Authors:** Georgia K. Chaseling, Katrina Blackett, Steve Vucic, Michael Barnett, Scott L. Davis, Ollie Jay, Nicole T. Vargas

**Affiliations:** 1 Thermal Ergonomics Laboratory, Heat and Health Research Centre, Faculty of Medicine and Health, University of Sydney, Camperdown, Sydney, NSW 2006 Australia; 2 Sydney Nursing School, Faculty of Medicine and Health, The University of Sydney, Sydney, NSW Australia; 3 Westmead Clinical School, University of Sydney Clinical School, Westmead, NSW Australia; 4 Central Clinical School, Sydney Medical School, Faculty of Medicine and Health, The University of Sydney, Sydney, NSW Australia; 5 Applied Physiology and Sport Management, Southern Methodist University, Dallas, TX USA

**Keywords:** Cooling strategies, Demyelination, Heat intolerance, Heat sensitivity, Physical activity, Uhthoff’s phenomenon

## Abstract

**Purpose:**

Cold-water ingestion improves exercise capacity in the heat for people with multiple sclerosis (MS). Whether cold-water ingestion also mitigates heat-related MS symptoms is unknown. Ingesting fluid is also limiting for people with MS with impaired bladder function. Therefore, we tested the hypothesis that swilling or ingesting cold-water (7°C) compared to ingesting thermoneutral water (37°C) would mitigate the onset of perceived MS heat-related symptoms and thermal sensation in heat-sensitive people with MS during exercise in the heat.

**Methods:**

On three occasions, 13 heat-sensitive participants with MS (41 ± 12 y; 67 ± 12 kg; 1.7 ± 0.1 m; 33.3 ± 9.4 ml·kg^−1^·min^−1^) cycled at 40% VO_2max_ at 35 ± 1°C; 30 ± 2% RH until volitional exhaustion (maximum of 60 min). Every 15 min, participants ingested (7_IN_) or swilled (7_SW_) 7°C, or ingested 37°C (37_IN_) water. Thermal sensation, heat-related MS symptoms, rectal (*T*_re_), and mean skin (*T*_sk_) temperature were recorded throughout.

**Results:**

Thermal sensation was cooler in the 7_SW_ (*P* < 0.01) and 7_IN_ (*P* = 0.04) compared to the 37_IN_ trial, but heat-related symptoms (*P* = 0.57), fatigue (*P* = 0.90), Δ*T*_re_ (37_IN_: 0.74 ± 0.37°C; 7_IN_: 0.65 ± 0.38°C; 7_SW_: 0.67 ± 0.34°C; *P* = 0.38) and Δ*T*_sk_ (37_IN_: 1.61 ± 0.82°C; 7_IN_: 1.67 ± 0.78°C; 7_SW_: 1.64 ± 0.69°C; *P* = 0.91), were not different between trials. Nine participants completed 60 min of exercise in the 37_IN_ trial whereas 10 participants completed 60 min of exercise in the 7_IN_ and 7_SW_ trials.

**Conclusion:**

Swilling and ingesting 7°C water induces a cooler thermal sensation in heat-sensitive people with MS exercising in the heat but does not mitigate heat-related MS symptoms. The capacity to complete 60 min of exercise with cold-water ingestion and swilling were comparable.

## Introduction

Up to 80% of people diagnosed with multiple sclerosis (MS) experience Uhthoff’s’ phenomenon (Uhthoff 1889) characterized by a temporary worsening of MS symptoms and a rapid onset of fatigue with exercise and/or heat exposure. Although an increase in core temperature may play a role, it is evident that people with MS can experience a transient worsening of symptoms even in the absence of a rise in core temperature (Chaseling et al. 2018; Davis et al. 1970; Nelson et al. 1958).

Exercise is recommended for people with MS as a disease modifying therapy (Motl and Pilutti 2016), and for mitigating MS-related fatigue (Motl and Pilutti 2012). People with MS have a reduced capacity to exercise in neutral (25°C) (Allen et al. 2019), warm (30°C) (Chaseling et al. 2020, 2018) and hot (35°C) (Chaseling et al. 2020) environments, compared to age- and fitness-matched controls, likely due to Uhthoff’s’ phenomenon. A transient worsening of symptoms has been reported to result in people with MS avoiding exercise (Kayes et al. 2011). However, few studies have investigated the efficacy of cooling strategies that can be used *during* exercise, particularly in hot environments, that mitigate heat-related MS symptoms and fatigue and improve exercise capacity. Our group previously demonstrated (Chaseling et al. 2018) heat-sensitive MS participants increase their exercise time-to-exhaustion in a warm environment (30°C, 30% relative humidity [RH]) by ~ 30% when ingesting very cold (1.5°C) compared to thermoneutral (37°C) water. This increase was achieved despite similar rises in core and skin temperature with cold-water ingestion at a comparable exercise time point to the thermoneutral water. Furthermore, a longer exercise time with 1.5°C water ingestion resulted in a greater end-exercise change in core temperature (+ 0.40°C vs + 0.26°C) compared to 37°C water ingestion. This observation suggests that perceptual factors such as changes in thermal sensation or rating of perceived exertion were positively influenced by cold-water ingestion thus altering exercise capacity, however perceptual data were not obtained to test this hypothesis (Chaseling et al. 2018).

A limitation of *ingesting* cold-water as a cooling strategy is that people with MS can experience bladder dysfunction (Zecca et al. 2016), which can become worse with heat exposure (Flensner et al. 2011). An alternative to ingesting, may be to *swill* water in the mouth. Swilling cold water stimulates cold-afferent thermoreceptors within the oral cavity (Morris et al. 2014) and has been shown to improve aerobic performance of healthy athletes without differences in core temperature (Burdon et al. 2013). However, no previous study has investigated the effect of swilling cold water on mitigating heat-related MS symptoms or exercise capacity in people with MS.

The primary aim of this study was to investigate whether swilling or ingesting cold water (7°C) compared to ingesting thermoneutral water (37°C) can mitigate heat-related fatigue and symptoms, and augment perceptions of thermal sensation while cycling in a hot (35°C, 30% RH) environment. A secondary aim of this study was to assess if exercise duration would be reduced with thermoneutral water ingestion, and whether an increase in exercise duration with swilling or ingesting cold water, if apparent, was due to changes in heat-related symptoms and/or thermal sensation. It was hypothesized that 1) both swilling and ingesting cold water would attenuate increases in thermal sensation, perceived heat-related fatigue and symptoms compared to thermoneutral water ingestion and 2) a longer exercise duration would be observed both with swilling and/or ingesting cold water compared to the thermoneutral water ingestion during exercise in a hot environment.

## Materials and methods

### Participants

Thirteen people with MS with self-reported heat sensitivity (relapsing–remitting; *n* = 12, secondary progressive; *n* = 1) and expanded disability status scale < 5 (0: normal neurological function, 5: able to walk without aid for 200 m), participated in this study (Table 1). A sample size of 10 participants was determined using a power calculation (G*Power 3 software Heinrich-Heine-Universität Düsseldorf, Germany) employing an α of 0.05, β of 0.80 and an effect size of 1.04, based on exercise time with very cold (1.5°C) compared to thermoneutral (37°C) water ingestion for people with MS (Chaseling et al. 2018). Because we anticipated a slightly smaller effect size with warmer water (7°C) compared to our previous research (1.5°C), 13 people were recruited for this study. All participants were non-smokers, had no history of cardiovascular, metabolic, or respiratory disorders, were not currently taking competitive muscarinic receptor antagonists, had not experienced symptom exacerbations in the past 3 months. This study was approved by the University of Sydney Human Research Ethics Committee (HREC No. 2016-214). All participants were informed of any risks involved with the study and provided their written informed consent.

**Table 1:** Participant characteristics and baseline outcome variables

Participant characteristics		
Sex	9 F/4 M	
Age, *y*	41 ± 12	
Weight,* kg*	67.0 ± 12.3	
Height, *m*	1.7 ± 0.1	
VO_2_ peak, *ml·min*^*−1*^*·kg*^*−1*^	33.3 ± 9.4	
Disease duration, *years*	8 ± 7	
Medication (*n out of 13)*	Tecfidera (3) Fingolimod (3) Natalizumab (2) Beta Interferon (1)	Baclofen (1) Ocrelizumab (1) No medication (1) Copaxone (1)
Perceived heat-related symptoms (*n out of 13)*	Fatigue (12) Leg weakness (5) Impaired vision (4) Tingling (3)	Cramping (3) Dizziness (1) Foot numbness (1) Spasticity (1)

### Study design

Trials were performed across the entire year at the Thermal Ergonomics Laboratory, University of Sydney. Participants completed one preliminary, and three experimental trials conducted in a randomized counterbalanced order and separated by a minimum of 48 h and a maximum of 2 weeks. Participants were not blinded to the trial condition. Participants were asked to withhold from ingesting caffeine, alcohol, and participating in strenuous exercise 12 h prior to testing and consume a light meal and ~ 500 mL of water 2 h prior to testing.

### Preliminary protocol

Participants completed a MS-specific medical history and lifestyle questionnaire followed by anthropometric measurements. Participants were familiarized with equipment, the volume and technique of swilling water and self-nominated up to three symptoms exacerbated by heat exposure and/or exercise, which were documented for subsequent trials. This session was concluded with a VO_2max_ test, involving 3 min of rest before commencing semi-recumbent cycling (Corival Recumbent; Lode BV, Groningen, the Netherlands) in a temperate (20°C, 30% RH) environment, at a resistance of 30 W, which increased 10 W every minute until volitional fatigue, or a plateau in their VO_2_ was observed (Heine et al. 2014).

### Experimental protocol

On arrival, participants self-inserted a rectal thermistor, followed by full instrumentation, then entered an environmental chamber maintained at 35 ± 1ºC, 30 ± 2% RH, where baseline measures were recorded for 15 min. Next, participants cycled on a semi-recumbent ergometer for 60 min at 40% of their VO_2max_, a workload that was chosen to replicate that of our previous study (Chaseling et al. 2018). Airflow was provided throughout by three 46-cm mechanical fans stacked vertically to generate a mean air velocity of 0.2 m·s^−1^. Every 15 min after the commencement of exercise, participants were given 3.2 ml·kg^−1^ of either 37°C or 7°C water to fully ingest without swilling or 7°C water to swill around the mouth without ingesting. The swill was administered in four or five equal volume aliquots. Participants were instructed to swish the water around in their mouth for 20 s, for a total time of 80–100 s and spit the fluid out after each swill. Participants reported thermal sensation and symptom severity 1 min before, one and 6 min after water administration. Ratings of perceived exertion (RPE) were taken using a standard Borg scale, before, and 6 min after each water administration. Following the cessation of exercise, the participant was weighed, provided cold water and remained in the environmental chamber at 18°C until any temporary symptom worsening was reversed.

### Instrumentation and measures

*Perceptual measures*: Thermal sensation was obtained using a 200-mm visual analogue scale (very cold: 0 mm, neutral: 100 mm, very hot: 200 mm) (Bright et al. 2019). Symptoms were measured using a scale marked 0–10 (0: no symptom, 5: moderate, 10: stop exercise). In the absence of a validated symptom scale for people with MS, the current symptom severity scale was developed by our team of researchers, based on the Borg scale, and research by Raccuglia et al. (Raccuglia et al. 2017, 2018). RPE was measured using the 6–20 Borg scale (6: no exertion at all, 11: somewhat hard, 17: very hard, 20: Maximal exertion) (Borg 1982). To assess the influence of water temperature/administration on heat-related symptoms, symptom scores were calculated as the sum of reported scores for each nominated symptom (i.e., on the symptom scale of 0–10), and presented as the change from baseline during exercise. Fatigue, being the most reported symptom of MS heat sensitivity, was analysed separately (Vargas et al. 2021). Average trial data are reported for thermal sensation, RPE, perceived heat-related fatigue and heat-related symptoms. Both average trial and end-exercise data are matched for time corresponding to the shortest trial duration for each participant when 60 min of exercise was not completed.

*Physiologic measures*: Rectal temperature (*T*_re_) was measured with general pediatric thermistors (TM400, Covidien, Mansfield, Massachusetts, USA), self-inserted to a depth of ~ 12 cm beyond the anal sphincter. Mean skin temperature (*T*_sk_) was calculated as the average temperature from the chest 30%, shoulder 30%, thigh 20%, and calf 20% (Ramanathan 1964), measured on the left side of the body using thermistors integrated into heat flow sensors (Concept Engineering, Old Saybrook, CT). All thermometric measurements were sampled every 5 s (NI cDAQ-91722 module, National Instruments, Texas, USA) and displayed in real-time using LabView (v7.0, National Instruments) (Chaseling et al. 2019, 2020). Body mass was measured on a platform scale (Mettler 1D1 Multirange; Germany) and calculated as the difference in pre- and post-exercise weight, accounting for fluid intake. Whole-body sweat rate (WBSR) was calculated by dividing the whole-body sweat loss (in grams) by the total exercise duration (in minutes). Heart rate was obtained from lead II of a 6-lead wireless electrocardiography (Quark T12x Asia Pacific PTY, Sydney Australia).

### Statistical analyses

All data are presented as mean and standard deviation (±). A one-way repeated measures ANOVA was used to analyze all data employing the independent variable of water temperature/administration method (3 levels: 37_IN_, 7_IN_, 7_SW_). Dependent variables were as follows: Baseline thermal sensation, RPE, heat-related fatigue and symptom severity, *T*_re_, *T*_sk_, and heart rate; Exercise duration, and RPE, thermal sensation, heat-related MS symptoms averaged throughout exercise; the change from baseline in *T*_re_, *T*_sk_ and heart rate, the change in *T*_re_ at symptom onset and all other thermometry and cardiovascular variables at the time of exhaustion, matched to the shortest duration trial. Statistical significance was set at an alpha level of 0.05, and the probability of a type 1 error was maintained at 5% for all post hoc comparisons using a Bonferroni correction. Eta-squared (η^2^) was used to calculate effect sizes using the ratio of the sum of squares of the effect and the total sum of squares (small η^2^ = 0.01), medium (η^2^ = 0.06), and large (η^2^ = 0.14) (Lakens 2013). All analyses were performed using GraphPad Prism (Version 9.2.0, GraphPad Software Inc., La Jolla, CA).

## Results

All data represent *n* = 13 except for heat-related fatigue (*n* = 12) because one participant did not nominate fatigue as a symptom; heat-related symptoms (not including fatigue) (*n* = 11) because two participants nominated *only* fatigue as a symptom. All baseline data are presented in Table 2. There were no differences in any baseline variables between conditions (all *P* > 0.05).

**Table 2:** Baseline outcome variables

Baseline Outcome Variables (mean ± SD)				
	*37*_*IN*_	*7*_*IN*_	*7*_*SW*_	*P-value*
Rectal temperature (°C)	36.9 ± 0.4	37.0 ± 0.3	36.9 ± 0.4	0.53
Skin temperature (°C)	34.8 ± 1.0	34.7 ± 0.9	34.9 ± 0.8	0.59
Heart rate (bpm)	80 ± 12	83 ± 16	79 ± 13	0.41
Thermal sensation (mm)	128 ± 19	119 ± 18	121 ± 23	0.24
RPE (a.u.)	6 ± 0	6 ± 0	6 ± 0	0.99
Fatigue score (a.u.)	1 ± 2	1 ± 2	1 ± 1	0.81
Symptom score (a.u.)	2 ± 2	1 ± 3	1 ± 2	0.70

*RPE* rating of perceived exertion, a.u: arbitrary units

### Thermal sensation, RPE, and heat-related MS symptoms

**Fig. 1:**
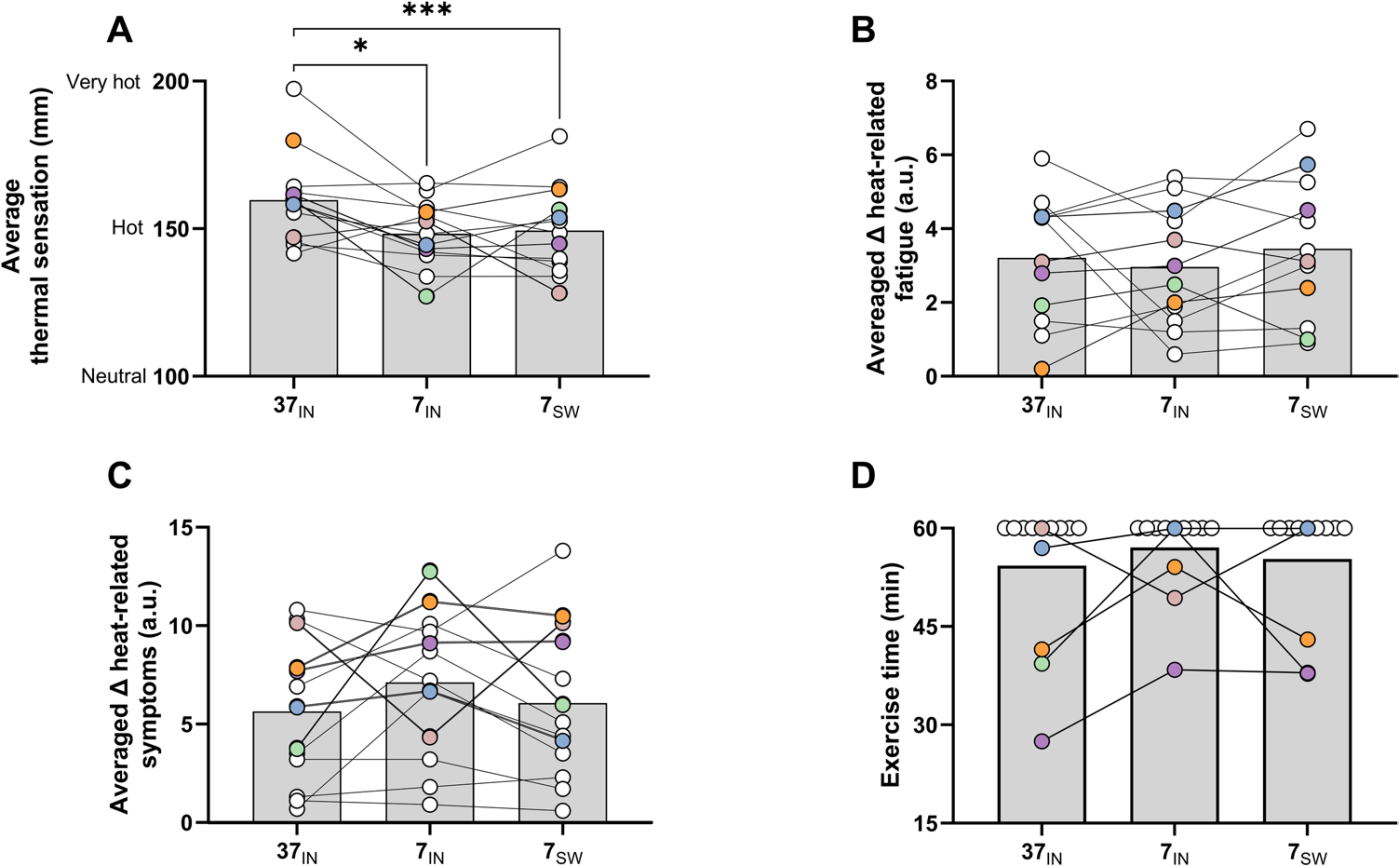
Group mean and individual data for average trial thermal sensation (**A**), average trial heat-related fatigue (**B**), average trial heat-related symptoms (**C**) and exercise time (**D**) during the 37_IN_, 7_IN_ and 7_SW_ trials. Coloured data represent participants who did not complete 60 min exercise in at least one out of the three trials. Single asterisk denotes statistical difference of *P* < 0.05; Triple asterisk denotes statistical difference of *P* < 0.001

Thermal sensation (100 mm = neutral, 150 mm = hot, 200 mm = very hot) was lower (i.e., participants felt cooler) in both the 7_SW_ (150 ± 15 mm; *P* < 0.001) and 7_IN_ (148 ± 11 mm, *P* = 0.04, η^2^ = 0.13) trials compared to the 37_IN_ (160 ± 15 mm) trial (Fig. 1A). However average RPE (14 = hard; 37_IN_: 14 ± 2; 7_IN_: 14 ± 2; 7_SW_: 14 ± 2; *P* = 0.69, η^2^ = 0.001), perceived heat-related fatigue (37_IN_: 3 ± 2; 7_IN_: 3 ± 1; 7_SW_: 3 ± 2; *P* = 0.52, η^2^ = 0.01, Fig. 1B) and perceived heat-related symptoms (37_IN_: 6 ± 4; 7_IN_: 7 ± 4; 7_SW_: 6 ± 4; *P* = 0.27, η^2^ = 0.03, Fig. 1C) were not different between trials.

### Exercise duration

Nine out of thirteen participants completed the full 60 min of exercise in the 37_IN_ trial, whereas 10 out of 13 participants completed 60 min of exercise in the 7_SW_ and 7_IN_ trial (Fig. 1D). From the four participants who could not complete 60 min of exercise in the 37_IN_ trial (~ 41 ± 12 min), all exercised for longer in the 7_IN_ (~ 53 ± 10 min) and the 7_SW_ trial (~ 45 ± 10 min). Nevertheless, no significant differences were observed between trials for overall exercise duration (37_IN_: 54 ± 11 min; 7_IN_: 57 ± 6 min; 7_SW_: 55 ± 9 min; *P* = 0.29, η^2^ = 0.02). The absolute workload (37_IN_: 56 ± 25 W; 7_IN_: 55 ± 26 W; 7_SW_: 55 ± 26 W; *P* = 0.26) did not differ between trials.

### Body temperatures, heart rate, and whole-body sweat rate

**Fig. 2:**
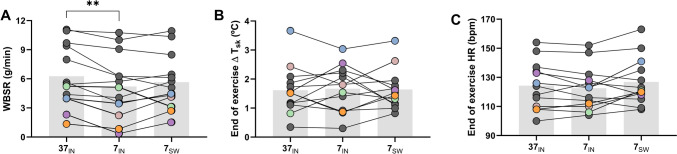
Group mean and individual data at the end of exercise in the 37_IN_ trial compared with the same time point in the 7_IN_ and 7_SW_ trials. Data are shown for whole-body sweat rate (WBSR; A), the change in skin temperature (*T*_sk_; B) and heart rate (HR; C). Dark gray circles represent participants who completed 60 min of cycling. Colored circles in graph A, B and C represent individual participants who did not complete 60 min exercise in at least one of out of the three trials. Double asterisk denotes statistical difference of *P* < 0.01

**Fig. 3:**
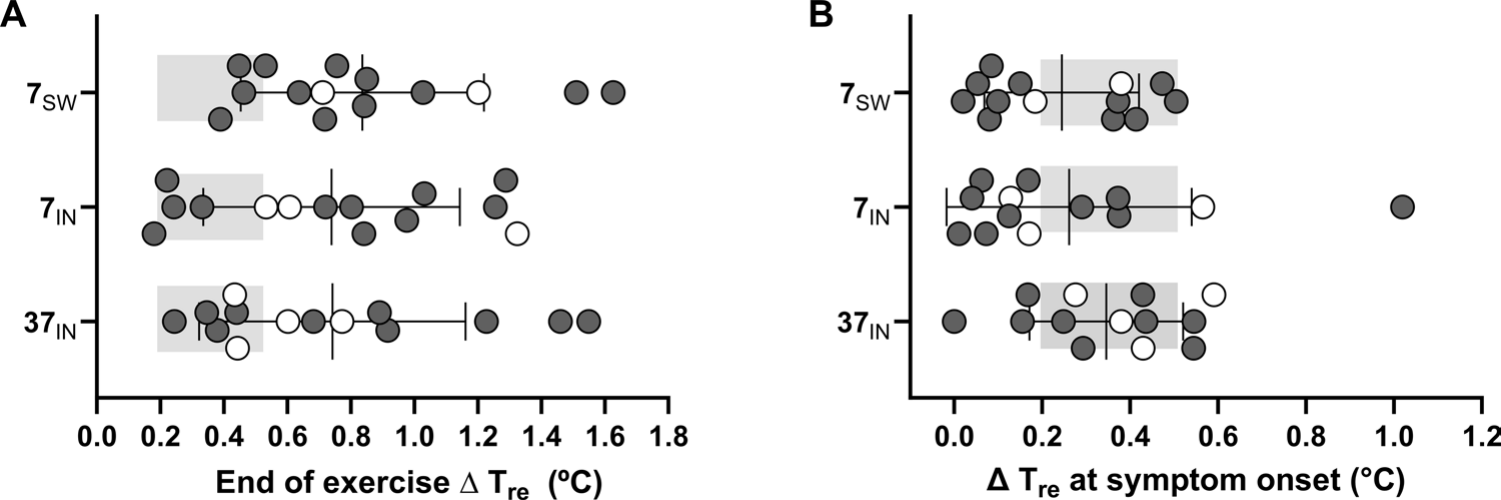
Group mean and individual data for the end of exercise rectal temperature (*T*_re_; **A**) and the change in rectal temperature when the first MS symptoms was recorded (**B**). White circles represent individual participants who did not complete 60-min exercise in at least one of out of the three trials. The gray shading represents the change in rectal temperature at which Uhthoff’s phenomenon is reportedly induced

Whole-body sweat rate was lower (*P* = 0.03, η^2^ = 0.02) in the 7_IN_ trial (6.3 ± 3.2 g⋅min^−1^) compared to the 37_IN_ trial (5.2 ± 3.4 g⋅min^−1^), but not the 7_SW_ trial (5.7 ± 2.8 g⋅min^−1^; Fig. 2A). The change from baseline to the end of exercise in *T*_re_ (37_IN_: 0.74 ± 0.37°C; 7_IN_: 0.65 ± 0.38°C; 7_SW_: 0.67 ± 0.34°C; P = 0.38, η^2^ = 0.01) and *T*_sk_ (37_IN_: 1.61 ± 0.82°C; 7_IN_: 1.67 ± 0.78°C; 7_SW_: 1.64 ± 0.69°C; *P* = 0.91, η^2^ = 0.0006) were not different between trials (Fig. 2B and 3B). End-exercise heart rate (37_IN_: 124 ± 16 bpm; 7_IN_: 123 ± 15 bpm 7_SW_: 127 ± 16 bpm; P = 0.18, η^2^ = 0.01) was similar between trials (Fig. 2C) and the change in *T*_re_ at which heat-related symptoms increased (Fig. 3A) was not different between trials (37_IN_: 0.34 ± 0.18°C; 7_IN_: 0.26 ± 0.2°C; 7_SW_: 0.24 ± 0.18°C; *P* = 0.45).

## Discussion

This study compared the effect of cold-water swilling and ingestion during exercise in the heat on thermal sensation, perceptions of heat-related fatigue, MS-related symptoms, and exercise capacity in heat-sensitive people with MS. Our main finding was that swilling and ingesting 7°C water improved thermal sensation (i.e., participants felt cooler; Fig. 1A). This finding was despite a lower sweat rate in the 7_IN_ trial only, with similar rises in core and skin temperature, and perceptions of heat-related fatigue (Fig. 1B) or MS symptoms (Fig. 1C) were not different compared to 37°C water ingestion. Our secondary finding was that while 4 out of 13 participants were not able to complete 60 min of exercise when ingesting 37°C water, and all of four these participants cycled for longer when ingesting or swilling 7°C water. These data demonstrate that (1) improvements in thermal sensation with cold-water swilling and ingestion are not paralleled by reductions in heat-related fatigue or MS symptoms, (2) people with MS experienced heat-related MS symptoms for the same rise in core temperature between trials, despite feeling cooler with swilling or ingesting 7°C water; and (3) exercise capacity in the heat may be improved with cold-water swilling or ingestion, however 9 of 13 participants exercised for a complete 60 min in the current study, so its efficacy is unclear.

### The effect of cold water on thermal perception, heat-related MS symptoms, and fatigue during exercise in the heat

Few studies have assessed the efficacy of cooling strategies during exercise to improve heat-related fatigue and MS symptoms. Cooling strategies that attenuate the increase in core temperature, or improve thermal perception, may also mitigate the onset of heat-related fatigue and symptoms. However, our findings demonstrate that while swilling or ingesting 7°C water induces a cooler thermal sensation despite a similar rise in core and skin temperature, perceived heat-related fatigue or MS symptoms during exercise in the heat are not altered. Similar findings were reported by Vargas et al. (Vargas et al. 2021), where reducing skin temperature by ~ 2°C led to an improved thermal sensation (patients felt cooler) at the end of 40 min of exercise, without differences in perceived heat-related fatigue, MS symptoms or core temperature compared to a control condition (Vargas et al. 2021). Furthermore, despite 9 of 13 participants completing 60 min of exercise during the 37_IN_ trial in the current study, 4 of the 13 stopped exercising due to volitional exhaustion. Of these four participants, heat-related MS symptoms (Fig. 1C) were worse during the 7_IN_ and 7_SW_ trials compared to the 37_IN_. In addition, for the 1 participant who exercised for longer in the 37_IN_ compared to the 7_IN_ trial, their heat-related symptoms were worse in the 37_IN_ compared to 7_IN_ (Fig. 1C; light pink circles). This finding suggests that people with MS were able to exercise for longer because they felt cooler, despite demonstrating worse perceived MS symptoms. Where the critical factor of Uhthoff’s phenomenon is a rise in core temperature of 0.2–0.5°C, employing cooling strategies that augment core temperature and not just thermal sensation is vital. This distinction is noteworthy because many individuals may use relatively ineffective cooling methods that merely create a sensation of coolness without adequately reducing core temperature. Our data indicate that such cooling strategies may be inadvisable, as methods that induce a subjective feeling of coolness do not necessarily contribute to the reduction of core temperature.

It is known that exercise and/or heat exposure cause a temporary worsening of symptoms in MS patients, yet there is little evidence that supports a direct relationship between a rise in core temperature and the onset of heat-related symptoms. Findings from the current study demonstrate a worsening of MS-related symptoms for the same rise in core temperature across all trials, despite feeling cooler during the 7_IN_ and 7_SW_ trial. However, nearly all participants experienced symptoms before a rise in core temperature of 0.2–0.5°C (Fig. 2E), which is the theoretical lower limit rise in core temperature to induce heat-related MS symptoms (Davis and Jacobson 1971). This is supported by findings from Nelson et al. (Nelson and McDowell 1959) who demonstrated in the absence of a rise in core temperature, sitting in a hot bath induced a transient worsening of symptoms in MS patients. Similar findings were observed by Poh et al. (Poh et al. 2017) who showed a worsening of postural sway in people with MS exposed to hot environments, independently of changes in core temperature. Together these findings suggest that a temporary worsening of heat-related MS symptoms may not be individually driven by a rise in core temperature, and that skin temperature or anticipatory factors may also play a role (Chaseling et al. 2020; Marino 2009).

### The effect of cold-water swilling and ingestion on exercise capacity in MS

Our research team has previously demonstrated people with MS could only exercise for ~ 33 min in a 30°C environment when ingesting 37°C water (Chaseling et al. 2018). This exercise time was increased by ~ 30% when MS participants drank 1.5°C water instead. The increased exercise time occurred despite greater rises in core temperature at the end of exercise with the 1.5°C, compared to the 37°C water ingestion. This study suggested that stimulation of cold-afferent receptors (Burdon et al. 2013) could improve exercise duration, without reductions in core temperature. It was anticipated in the current study that exercise in a hotter environment (i.e., 35°C vs 30°C) would exacerbate heat-related MS symptoms and fatigue, leading to a reduced exercise capacity when ingesting 37°C water. Despite self-reported confirmation of heat-sensitivity we cannot exclude the possibility that participants in the present study had a greater overall tolerance to exercise in the heat. It is possible that if a greater sensitivity to the heat or exercise intolerance were observed during the 37_IN_ trial, or a different exercise protocol was employed, such as a time trial, or a fixed RPE protocol, an effect of either the swilling or ingesting may have been observed. Indeed, participants (4 of 13) that could not complete 60 min of exercise during the 37_IN_ trial, all exercised for longer with cold-water ingestion and swilling. It therefore seems likely that the lack of effect of cold-water ingestion/swilling on exercise duration was a result of a fixed-intensity and limited-duration exercise protocol. Nevertheless, the primary aim of this study was to assess differences in heat-related symptoms, fatigue and thermal perception with cold-water swilling and ingestion with a fixed exercise stimulus.

### Thermoregulatory responses of people with MS to exercise in the heat

Early research on Uhthoff’s phenomenon in animals models suggested that a rise in core temperature of 0.5°C of a demyelinated nerve reduces its conduction velocity by a greater amount compared to a myelinated nerve which is what could lead to a worsening of symptoms for heat-sensitive people with MS (Davis & Jacobson 1971). Research has also suggested that demyelination of nerves leads to thermoregulatory dysfunction for people with MS, causing a greater rise in core temperature compared to healthy controls. Our research team has previously shown that compared to healthy controls, people with MS have a delayed onset of sweating (Chaseling et al. 2020) and blunted sweat rates (Allen et al. 2019; Chaseling et al. 2020; Vargas et al. 2021) during exercise in the heat. However, this blunted sweat response does not lead to greater rises in core temperature (Allen et al. 2019; Chaseling et al. 2020). Irrespective of whether participants could complete 60 min of cycling or not, most participants exercised beyond a rise in core temperature of 0.5°C (Fig. 2D). This was despite participants demonstrating a worsening of their heat-related fatigue and MS symptoms prior to a rise in core temperature of 0.2–0.5°C (Fig. 2E). Our group has previously demonstrated similar findings where participants could exercise in warm and hot environments (25°C, 30°C and 35°C) beyond a rise in core temperature of 0.5°C (Allen et al. 2019; Chaseling et al. 2020). Collectively, these data demonstrate that despite an obvious transient worsening of heat-related symptoms and fatigue, people with MS can exercise above a rise in core temperature of 0.2–0.5°C, which is an important consideration given the profound physical and psychologic benefits of exercise for people with MS.

Our previous research has shown in adults without MS, ingesting cold water reduces sweat production which is offset by a parallel reduction in internal heat storage (Morris et al. 2014). This reduced sweat output is likely mediated by afferent visceral thermoreceptors that modify the central drive for sweat production. A reduced whole-body sweat rate in the 7_IN_ trial compared to the 37_IN_ suggests that people with MS have a similar afferent response to people without MS with cold-water ingestion (Fig. 2C) which seem to offset any internal heat loss resulting in a similar rise in core temperature between trials (Fig. 2A).

### Considerations

There is no gold-standard for obtaining objective measures of fatigue in clinical populations, especially in MS. This study utilized an exercise time-to-exhaustion protocol with a fixed end point of 60 min. This protocol was selected to replicate one that was previously and successfully used in our laboratory (Chaseling et al. 2018. Time to exhaustion trials have been used to assess exercise performance in the heat in healthy adults (González-Alonso et al. 1999), however, because of the low relative intensity used in the current study, it is possible that a different study design such as a time trial (Racinais et al. 2015) or fixed RPE protocol (Schlader et al. 2011) may have elicited different results. However, our exercise protocol combined with the environmental conditions was still sufficient to induce a moderate level of heat stress and development of heat-related fatigue and MS symptoms, yet most participants exercising for 60 min in the heat limited the efficacy of the cold-water swill and ingestion. Alternative protocols may be beneficial to distinguish the amount of work completed within a given time. However, like the current study, if exercise duration is too short, differences in outcome variables may not emerge. A control group was not used in this study, so it is unclear if there would be differences in thermal sensation and perceived exertion between MS and healthy controls. From a thermophysiological perspective, our laboratory has demonstrated that resting (Chaseling et al. 2019) and the change in core and skin temperature at the end of exercise (Chaseling et al. 2020, 2018) is not different between MS and control participants. Given MS heat-related symptoms are specific to this cohort, the MS participants served as their own controls, focusing on within-group responses rather than between-group differences. Current exercise in the heat guidelines for people with MS suggest exercising in a cool or shady place, take additional breaks, and drink cold/icy water (Australia.). Our laboratory’s previous findings (Chaseling et al. 2018) and findings from the current study support the recommendation that drinking cold (1.5°C) or cool (7°C) water can improve thermal sensation and may improve exercise tolerance in hot environment, however swilling cold water is not an effective cooling strategy.

## Conclusion

This study examined the impact of swilling or ingesting cold water on thermal sensation, heat-related fatigue, MS-related symptoms, and exercise capacity in heat-sensitive people with MS during exercise in the heat. Although cold-water swilling and ingestion produced a cooler thermal sensation, neither intervention improved perceptions of heat-related fatigue or associated symptoms. Importantly, all participants developed heat-related symptoms for the same rise in core temperature across trials, despite feeling cooler when swilling or ingesting 7°C water. Given that Uhthoff’s phenomenon is triggered by a core temperature increase of 0.2–0.5°C, future research should explore implementing cooling strategies that effectively lower core temperature rather than just thermal sensation.

## Data Availability

The datasets generated during and/or analyzed during the current study are available from the corresponding author on reasonable request.

## Abbreviations

ANOVA: Analysis of variance
RH: Relative humidity
*T*_sk_: Meant skin temperature
VO_2_: Volume of oxygen
W: Watts
MS: Multiple sclerosis
RPE: Rating of perceived exertion
*T*_re_: Rectal temperature
WBSR: Whole-body sweat rate
